# Viscosity-Augmented Percutaneous Sclerotherapy for Recurrent Thoracic Spinal Aneurysmal Bone Cyst: A Technical Innovation to Mitigate Venous Washout

**DOI:** 10.7759/cureus.103014

**Published:** 2026-02-05

**Authors:** Harsh Agrawal, Saktthi Shanmuganathan, Naresh Kumar, Anil Gopinathan

**Affiliations:** 1 Orthopaedic Surgery, National University Hospital, Singapore, SGP; 2 Interventional Radiology, National University Hospital, Singapore, SGP

**Keywords:** absorbable gelatin sponge, aneurysmal bone cysts, interventional radiology, local neoplasm recurrence, sclerotherapy, sodium tetradecyl sulfate, spinal neoplasm, thoracic vertebrae

## Abstract

A 27-year-old male was operated on for a thoracic seven-vertebra aneurysmal bone cyst (ABC). On the six-year follow-up, he had radiological signs of recurrence. Treatment plan for recurrence was multiple sessions of percutaneous intralesional sclerotherapy with 3% of sodium tetradecyl sulphate (STS). The remarkable vascularity of the lesion resulted in rapid washout of the sclerosant mixture. Henceforth, an improvised technique was used in the third attempt. A thick sclerosant mixture injectate consisting of 3 ml of 3% STS mixed with 2 ml of gelatin sponge slurry (EG Gel 350-650 micron) and 2 ml of computed tomography (CT) contrast was used. At the six-month follow-up, there was new bone formation along the margins of the lytic lesion. The patient received two more sessions of the same injectate. At the two-year follow-up, the lesion showed no expansion of the lytic area with further increase in sclerosis. Modification of the injection technique with the use of a thick sclerosant mixture reduced the washout time. This case report describes the easy reproducibility of the technique and the simple logical problem-solving method, which is fundamental to Interventional radiology.

## Introduction

Aneurysmal bone cyst (ABC) is a locally aggressive benign vascular lesion affecting the bones. The long bones are more frequently affected. Within the spine, these lesions are mostly encountered in the lumbar region [1-3]. It may present as a paravertebral mass, more commonly found along the posterior elements than in the vertebral body [4]. Management aims to prevent vertebral collapse, pain, and neurological complications. ABCs are usually treated by thorough curettage, either as a single or multiple stage procedure. Recurrence of ABCs after the initial excision is not uncommon (≥30%) [5,6].

We present a case of post-surgical recurrence of ABC in the thoracic spine that was progressively enlarging and was successfully treated through an improvised technique of percutaneous sclerotherapy.

## Case presentation

A 27-year-old male presented with an acute onset of bilateral lower limb numbness for four hours. He had been suffering from upper back pain for three weeks before the onset of numbness. There was no preceding event of trauma or any other significant history. Examination revealed deep tenderness over mid thoracic region and decreased sensations around and below the level of the T10 dermatome, while motor power and reflexes were normal.

Plain X-ray showed a well-defined expansile lytic lesion involving the 7th thoracic vertebra. MRI confirmed the findings, demonstrating multiple septations and fluid-fluid levels suggestive of an ABC. The lesion involved the posterior two-third of the body, left neural arch, and posterior elements, with some expansion into paravertebral muscles. Severe canal stenosis and flattening of the thoracic cord required urgent surgical intervention.

The patient underwent preoperative selective embolization of bilateral T7 intercostal arteries. Thereafter, T4 to T10 instrumented stabilization, spinal cord decompression with laminectomy, and curettage of T7 vertebra was carried out. The bone defect was filled with hemostatic agents: injectate of gelatin granules and human thrombin (Floseal, Baxter, Illinois, USA) and fibrillar oxidized regenerated cellulose (Fibrillar Surgicel, Ethicon, New Jersey, USA). Post-operative histopathology confirmed the lesion to be an ABC. He made a good post-operative recovery.

**Figure 1:**
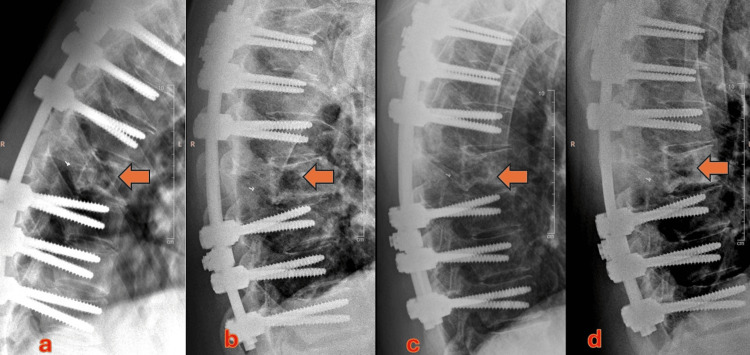
a) Before starting sclerotherapy; the arrow depicts the vertebra with lytic changes. b) Radiograph after two sessions of sclerotherapy with sodium tetradecyl sulfate (STS) foam showing not obvious improvement; the arrow depicts the area of lysis. c) Obtained one month after the first injection of STS + gelatin sponge slurry; shows early sclerotic changes within the previously seen lytic area, marked by an arrow. d) Obtained one month after three sessions of sclerotherapy with STS+ gelatin sponge slurry. A marked increase in the bony sclerosis within the lytic lesion is shown by the arrow.

The clinical and radiological progress was monitored through periodic clinical examination, radiographs, and computed tomography (CT) scans. Six years after the surgery, radiographs and CT scan showed an increase in size of the residual lytic areas within the T7 vertebral body (Figure 1a). Over the subsequent follow-ups, this started enlarging, although the patient was neurologically intact. Following a multi-disciplinary discussion, the patient was offered active management options to treat the enlarging recurrence of the ABC.

The patient was not keen on another surgery. We thereby explored non-surgical methods, opting for serial embolisation of the lesion. Angiographic interrogation of multiple segmental arteries and bronchial arteries showed vague capillary phase tumoral blush. There was no hypervascularity. Hence, there was no target for selective trans arterial embolisation. Thereafter, we offered him percutaneous interventional options viz, cryoablation with augmentation and serial sclerotherapy. The patient agreed to a few sessions of percutaneous sclerotherapy proposed to be done at 6-to-8-week intervals.

Injection technique

**Figure 2:**
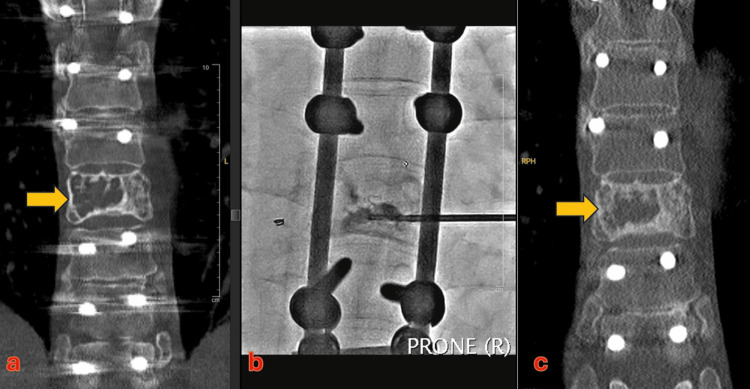
a) Coronal CT reconstructions  demonstrating the recurrent aneurysmal bone cyst (ABC), marked by an arrow. b) An intraprocedure radiograph during percutaneous transpedicular sclerotherapy. c) Note the marked increase in the sclerosis within the previously seen lytic lesion (shown by the arrow) following sclerotherapy.

All the sessions of percutaneous sclerotherapy were performed with combined CT and fluoroscopy guidance under local anaesthesia and sedation (with midazolam and fentanyl). At the first session, a 17-G Bard Truguide needle was inserted into the lytic vertebral lesion through transpedicular access (Figure 2). Multiple septations were traversed to ensure access into most of the cyst within the vertebral body. The cyst was flushed with normal saline. Subsequently, foam sclerotherapy was performed with 3% of sodium tetradecyl sulphate (STS). Owing to the remarkable vascularity, the sclerosant mixture washed out of the lesion rapidly. A second session was planned in eight weeks. However, due to the COVID pandemic, the patient came for the next follow-up after 10 months. There was no obvious response to the first session of sclerotherapy on the CT scan. The technique and injectate used in the second session were similar to the first session. The technical deficiencies of the first two attempts resulted us in devising an improvisation in the sclerotherapy technique (Figure 1b).

In the next attempt at the eight-week interval, we improvised the technique. At this session, the modification was to "prolong the dwell time of sclerosant within the lesion." The main differences were as follows: i) a larger (13G Jamshidi) needle was introduced into the cyst through a transpedicular route; ii) instead of STS foam, we used an injectate consisting of 3 ml of 3% STS mixed with 2 ml of gelatin sponge slurry (EG Gel 350-650 micron) and 2 ml of CT contrast. This thick sclerosant mixture was injected until a uniform distribution of the contrast was seen on fluoroscopy. After the third injection, the patient was advised follow up at six months to confirm sclerosis before attempting other injections. Radiographic follow-up at six months showed improvement in the new bone formation along the margins of the lytic lesion. Hence, two more sessions of sclerotherapy were performed using the same technique eight weeks apart.

Radiographs at one month from the last injection showed a significant increase in the sclerosis within the ABC (Figures 1c, 1d). Radiographic follow-up over the next two years has shown a further increase in the sclerosis with no expansion of the lytic area. The patient has remained asymptomatic throughout.

## Discussion

ABCs are rare (1% of primary bone tumors), with 6-22% occurring in the mobile spine [7]. In cases with no neurological involvement, selective arterial embolization (SAE) is currently advocated as the first line of management [5,6]. SAE is also used as an adjunct to operative management to reduce intraoperative blood loss [8,9]. Surgery is strongly indicated in an aggressive tumor with neurological involvement. En bloc resection of the lesion is advised to prevent recurrence. However, in some cases, the location of the lesion can pose challenges in intraoperative accessibility, thereby preventing complete resection [7]. Alternative options include curettage of the lesion without instrumentation or minimally invasive techniques such as percutaneous cryoablation or intralesional injection of sclerotherapy agents [10-12]. The role of radiotherapy can be considered where the lesion is inaccessible or surgical management is an absolute contraindication [13,14]. The use of various agents such as sodium tetradecyl sulfate, polidocanol, doxycycline, and absolute alcohol can be found in the literature [15-18]. Table 1 summarizes the use of various sclerosing agents in ABCs and the recurrence rate.

**Table 1:** Summary of the use of various sclerosing agents in aneurysmal bone cysts and the recurrence rate

Year	Author	Age group	Number of patients	Procedure	Composition	Follow-up	Recurrence
2013 [14]	Brosjö O	3-26 years	38; including two sacral lesions	CT or fluoroscopy guidance	2–4 mg polidocanol per kg body weight; three injections at interval of four weeks; median 4 injection per patient	4 to 37 months (mean 17 months)	3% (1)
2013 [15]	Shiels WE 2nd	3-18 years	20; five spinal lesions	CT or fluoroscopy guided	Doxycycline biofoam (concentration of 10 mg/mL)	2 years	1
2015 [5]	Batisse F	3-7 years	19; including three spinal sites	CT (2) or fluoroscopy guided (17) percutaneous sclerotherapy	Ethibloc* (6), aetoxisclerol (9), liquid absolute alcohol (2), absolute alcohol gel (2)	2 years	None
2021 [16]	Dalili D	7-52 years	8	CT or fluoroscopy guided	3% sodium tetra-decyl sulfate	5 years	No recurrence; two patients had surgery for persistent neurodeficit
2021 [17]	Masthoff M	6-25 years	16; three spinal lesions	Trans-arterial embolization follow by fluoroscopic sclerotherapy	DiscoGel (96% ethyl alcohol, cellulose derivative product, and tungsten) and polidocanol	6.7–47.5 months (median 27.3 months)	No
2022 [12]	Wong M	3-24 years	14; cervical	CT guided	Doxycycline biofoam (concentration of 10 mg/mL)	1.5 to 5.9 years (median 29.5 months)	14% (2)

Our patient initially underwent SAE followed by excision and stabilization with instrumentation. The lesion recurred after six years. Based on the angiographic pattern, there was no role for embolisation. Surgery was deemed to be a high-risk endeavor, especially when the patient was asymptomatic. Hence, a minimally invasive technique was offered to arrest the enlargement of the recurrent lesion.

Sclerosants are extensively used in treating vascular malformations due to their ability to cause direct damage to the inner endothelial lining of the blood vessel, and result in thrombotic occlusion of abnormal vascular spaces. Since ABCs are intra-osseous vascular lesions, they are expected to work similarly by inciting an inflammatory response, followed by reactive scar formation in the walls of the cystic spaces. Sclerosing agents have been extensively used in managing ABC of the long bones and spine.

In our patient, one of the challenges was the rapid venous washout of the sclerosant when used as a foam, as seen from the poor response to the first two sessions. Hence, we improvised our technique by mixing it with a thick slurry of gelatin sponge. The higher viscosity of the mixture allowed a longer dwell time of the sclerosant within the ABC (by slowing the washout). After this simple improvisation of technique, there was dramatic improvement in the response. We selected gelatin sponge for increasing the viscosity of the sclerosant mixture because of its relative ease of availability, proven safety and extensive experience of using them in intravascular spaces, and their biodegradable nature. This technique is reproducible, with similar technical risk mitigation and spinal safety considerations akin to standard sclerotherapy technique; as it involves change in the injectate mixture and not the procedure. This case report acts as a potentially useful adjunct to standard sclerotherapy technique and further studies will help strengthen the scientific evidence for technical replication.

## Conclusions

This report reinforces the value of percutaneous sclerotherapy, an outpatient procedure in treating ABCs of the spine. Since angioembolization failed to treat the recurrent tumour, percutaneous sclerotherapy was conceived as an appropriate treatment alternative. Despite this tumour not having any feeding vessels, the high vascularity of the lesion resulted in accelerated venous washout of the sclerotherapy agent. A simple technical improvisation of mixing the sclerosant with gelatin sponge slurry increased the viscosity of the injectate. The improvised injectate mixture significantly improved the efficacy of the procedure, as demonstrated in our case example. This simple, innovative modification of the technique helped mitigate another potential surgical intervention for the patient.
